# A case of lactobezoar: Outpatient management in a neonate

**DOI:** 10.1002/jpr3.70061

**Published:** 2025-07-07

**Authors:** Shivani Kamal, Sayeh Akhavan, Joshua Warolin, Ayesha Baig

**Affiliations:** ^1^ Pediatric Residency Program Valley Children's Healthcare Madera California USA; ^2^ Pediatric Gastroenterology Valley Children's Healthcare Madera California USA

**Keywords:** bezoar, gastroenterology, vomiting

## Abstract

A lactobezoar is a conglomerate of undigested and partially digested milk components and is the most common form of bezoar in infants. Described treatments include hospitalization for intravenous fluids and cessation of feeds, endoscopy with administration of *N*‐acetyl cysteine, and surgical or endoscopic removal. We report a case of a 5‐month‐old infant with a history of prematurity of 28 weeks, with poor feeding and emesis, found to have a gastric lactobezoar on imaging without signs of obstruction that was managed without surgical intervention. This case presentation describes the successful treatment of a patient with 100% whey formula for the treatment of lactobezoar, avoiding invasive treatment and hospitalization.

## 1 INTRODUCTION

A bezoar is a collection of undigested material that accumulates in the gastrointestinal (GI) tract that can lead to obstruction. Specifically, a lactobezoar is a conglomerate of partially digested milk components and is the most common form of bezoar in neonates.^1^ Complications include vomiting, poor weight gain, respiratory distress, gastric outlet obstruction, and rarely, gastric perforation.^2^, ^3^ Described treatments include hospitalization for intravenous (IV) fluids and cessation of feeds, endoscopy with administration of *N*‐acetyl cysteine, and surgical or endoscopic removal.^3^ Outpatient treatments were difficult to identify, with no identified reports describing the use of whey‐predominant formula (higher whey:casein ratio) for treatment of a lactobezoar. We present the case of an infant with a lactobezoar who was managed outpatient with 100% whey formula, without the need for hospitalization and invasive treatment.

## 2 CASE REPORT

A 5‐month‐old infant with a history of prematurity of 28 weeks presented to his pediatrician with persistent emesis and poor feeding. Pertinent medical history included chronic lung disease and being an infant of a diabetic mother. He had a prolonged Neonatal Intensive Care Unit (NICU) stay after birth due to extreme prematurity, requiring intubation and later transitioned to room air. The infant was discharged home with feeding regimen of Neosure fortified to 22 kcal, able to feed by mouth without issue. He was in a good state of health for a few weeks after discharge from the NICU, tolerating his feeds without concerns for GI symptoms. However, he began to develop emesis at an increasing frequency in the week before presentation. The nonbloody, nonbilious emesis occurred with every feed, appearing like formula. It was large volume and was occasionally projectile. His mother changed the formula to Enfacare 1 week before presentation which did not help the symptoms. Associated symptoms included increased tiredness compared to baseline and intermittent respiratory symptoms with transient retractions that self‐resolved and were not associated with feeds or during emesis.

At his pediatrician, exam was notable for mild subcostal retractions, but his abdominal exam was reassuring with active bowel sounds and only slight distension. The infant appeared well hydrated with strong pulses, however, had some slight congestion. The infant had normal vitals and appropriate growth parameters for corrected gestation age, without any noted weight loss. Laboratory work reviewed from urgent care visit the day prior for concern of cough and emesis, which included a normal comprehensive metabolic panel without signs of electrolyte derangement or dehydration.

**Figure 1:**
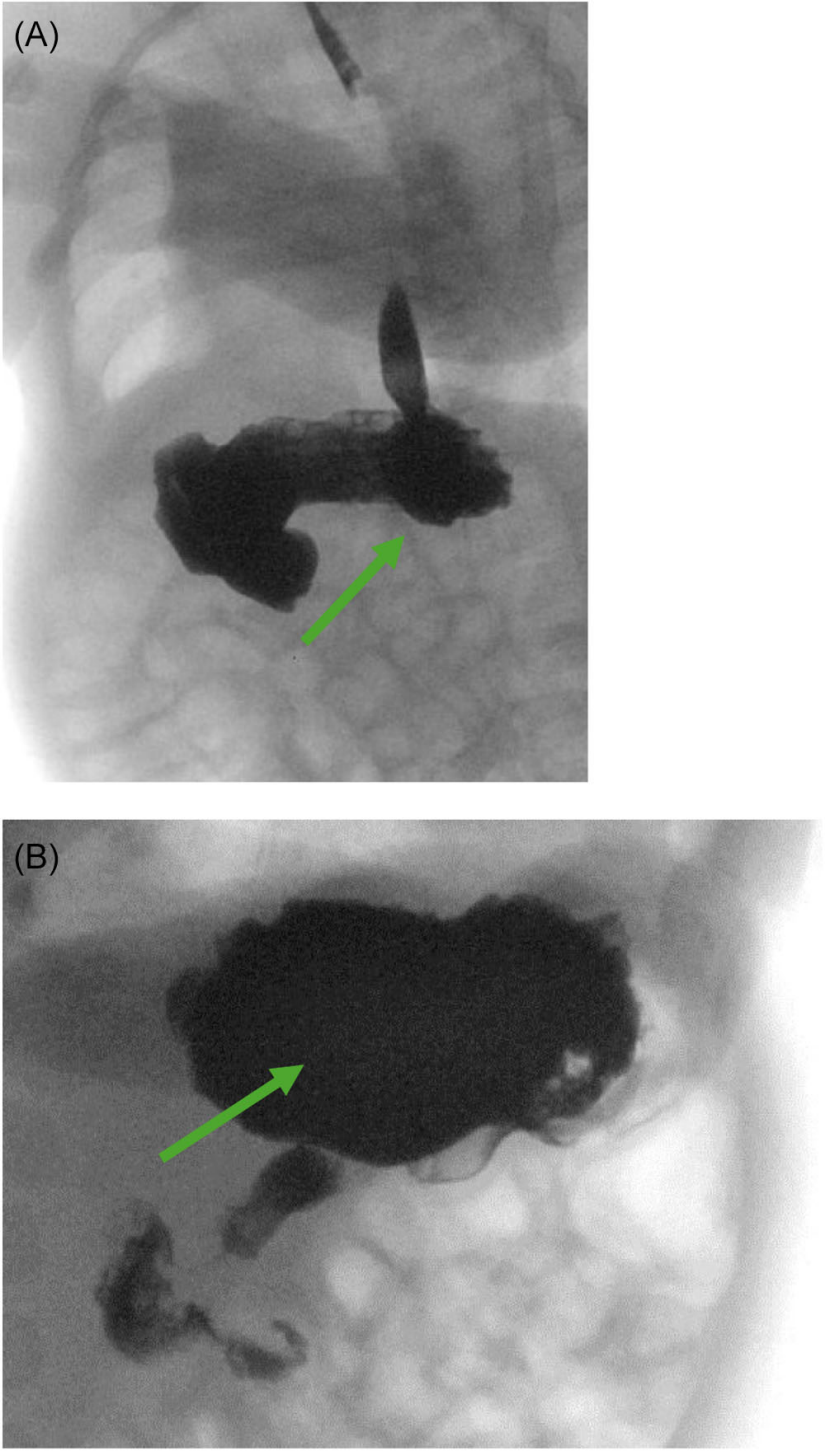
Abdominal X‐ray showing nonmobile mass, consistent with lactobezoar. (A) Upper GI series with KUB images showing non mobile mass, consistent with lactobezoar in the stomach. (B) Magnified view of the well‐defined, heterogeneous mass consistent with a lactobezoar (arrow).

**Figure 2:**
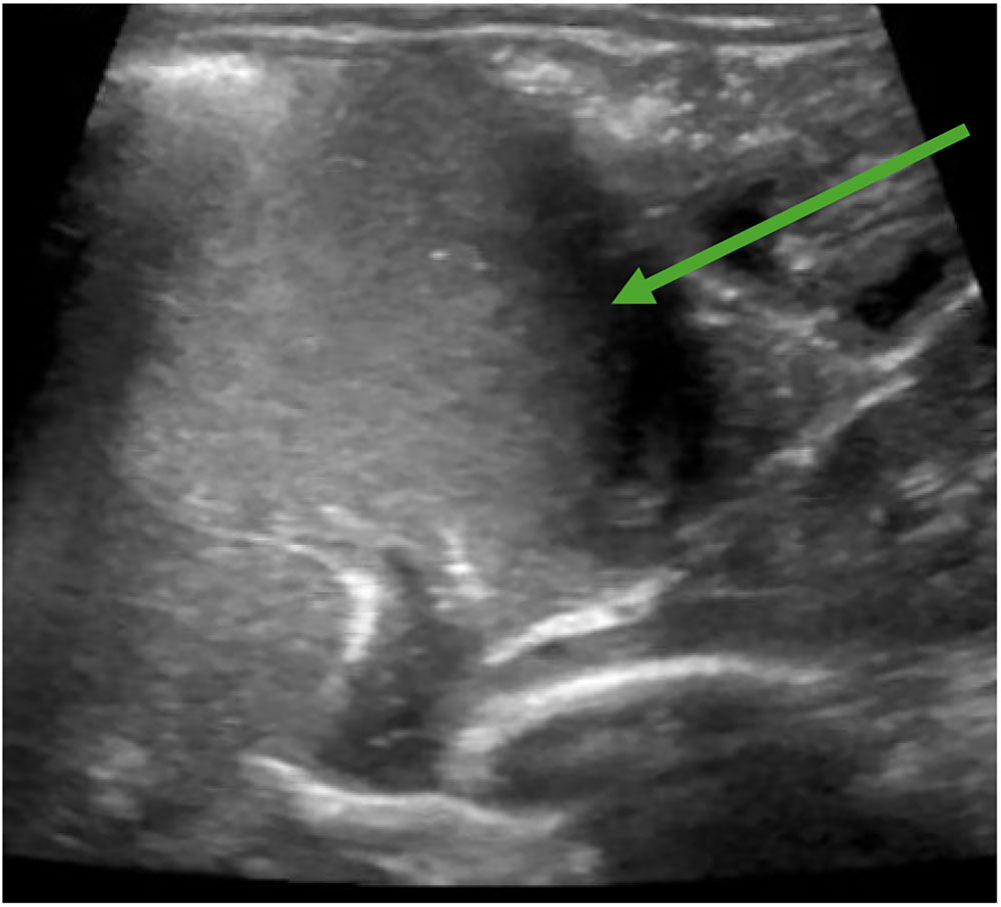
Stomach on abdominal ultrasound after treatment demonstrating no lactobezoar. Arrow indicating where prior lactobezoar present.

The pediatrician ordered a fluoroscopic upper GI series with abdominal X‐ray to assess for any anatomic abnormality given his history of chronic lung disease and emesis. The study incidentally showed a presumed lactobezoar in the stomach without signs of gastric outlet obstruction (Figure 1A,B). The esophagus, stomach, pylorus, and duodenum appeared normal in size with successful passage of barium contrast into the small bowel. After consultation with the on‐call pediatric gastroenterologist, his formula was changed to Gerber Extensive HA, a 100% whey‐based formula. At 1 week follow‐up he had resolution of emesis and was tolerating his feeds. Given the patient had history of chronic lung disease with multiple imaging studies done in past, the case was discussed with on call pediatric gastroenterologist and radiology to determine appropriate follow‐up imaging to minimize radiation exposure. In shared decision between the above providers, an abdominal ultrasound was ordered 2 weeks later which showed clear stomach contents moving without large solid elements or evidence of lactobezoar (Figure 2).

## 3 DISCUSSION

Lactobezoar, a collection of partially digested milk components, has the potential to cause poor health outcomes if not promptly identified and treated. Risk factors include premature birth, low birth weight, drinking fortified formula, and formula with higher casein concentration.^1^ Casein, a milk protein coagulates more regularly in the stomach than whey, a less dense and more acidic animal‐based protein. This can contribute to slower digestion and emptying, allowing milk and mucus to accumulate in the stomach leading to increased risk of lactobezoar formation. Interestingly, breast milk whey:casein ratios fluctuate but often have a 50:50 ratio, whereas cow's milk has a whey:casein ratio of approximately 20:80.^4^ Standard infant formula is often fortified with whey at a 60:40 whey:casein ratio; however, formula with higher whey concentrations (80:20 or solely whey formula) exists.^4^

While the fluoroscopic upper GI series with abdominal X‐ray originally ordered by the pediatrician was to evaluate for an anatomic abnormality, in general it is a highly effective tool to rapidly assess for a number of different potential etiologies of chronic vomiting in infants. Examples include gastric outlet obstruction or gross dysmotility. Given the rapid clinical improvement in the patient following formula change alone, it was felt that an abdominal ultrasound would be sufficient to determine if the lactobezoar had resolved without exposing the patient to the additional radiation from a repeat upper GI series study.

## 4 CONCLUSION

Symptoms of lactobezoar include vomiting, diarrhea, and respiratory distress. A review of the literature describes that lactobezoars may be underdiagnosed due to the nonspecific symptomology. Provider knowledge of lactobezoars may increase diagnosis of this condition. Management for patients with this diagnosis who present without emergent or acute findings is not well described. Current treatments for a lactobezoar described in the literature require hospitalization or are invasive.^3^ This case presentation describes the successful treatment of a patient with 100% whey formula for the treatment of lactobezoar which avoids invasive intervention. Formula with a higher whey:casein ratio than standard formula, or solely whey formula, may be considered as a first line treatment for lactobezoars in the outpatient setting.

## CONFLICT OF INTEREST STATEMENT

The authors declare no conflicts of interest.

## ETHICS STATEMENT

Written informed consent was obtained from the parent's legal guardian for the publication of case report.
